# Nocardioides agri sp. nov., isolated from garden soil

**DOI:** 10.1099/ijsem.0.006407

**Published:** 2024-06-18

**Authors:** Md. Amdadul Huq, Kihong NAM, Md. Shahedur Rahman, M. Mizanur Rahman, Md. Anowar Khasru Parvez, Kwon-Kyoo Kang, Shahina Akter

**Affiliations:** 1 Department of Food and Nutrition, Chung-Ang University, Anseong-si, Gyeonggi-do, 17546, Republic of Korea; 2 Department of Horticultural Life Science, Hankyong National University, Anseong-si, Gyeonggi-do, 17579, Republic of Korea; 3 Department of Genetic Engineering and Biotechnology, Jashore University of Science and Technology, Jashore 7408, Bangladesh; 4 Department of Biotechnology and Genetic Engineering, Faculty of Biological Science, Islamic University, Kushtia-7003, Bangladesh; 5 Department of Microbiology, Jahangirnagar University, Savar, Dhaka-1342, Bangladesh; 6 Department of Food Science and Biotechnology, Gachon University, Seongnam, 461-701, Republic of Korea

**Keywords:** 16S rRNA, genome sequence, Gram-stain-positive, *Nocardioides agri*

## Abstract

A novel bacterial strain, designated as MAH-18^T^, was isolated from soil sampled in a flower garden. Cells of strain MAH-18^T^ were Gram-stain-positive, aerobic, motile, and rod-shaped. The colonies were beige in colour, smooth, and spherical when grown on Reasoner's 2A agar medium. Strain MAH-18^T^ grew at 20–40 °C, pH 6.0–8.0, and 0–1.0 % NaCl. Cells were able to hydrolyse aesculin, gelatin, and Tween 20. According to the 16S rRNA gene sequence comparisons, the isolate was determined to be a member of the genus *Nocardioides* and most closely related to *Nocardioides pyridinolyticus* OS4^T^ (97.9 %), *Nocardioides hankookensis* DS-30^T^ (97.9 %), *Nocardioides aquiterrae* GW-9^T^ (97.6 %), *Nocardioides soli* mbc-2^T^ (97.5 %), *Nocardioides conyzicola* HWE 2-02^T^ (97.4 %), and *Nocardioides mangrovi* GBK3QG-3^T^ (96.3 %). Strain MAH-18^T^ has a draft genome size of 4 788 325 bp (eight contigs), 4572 protein-coding genes, 46 tRNA, and three rRNA genes. The average nucleotide identity and digital DNA–DNA hybridization values between strain MAH-18^T^ and the closest type strains were 81.5–83.4 % and 24.4–25.8 %, respectively. *In silico* genome mining revealed several biosynthetic gene clusters in the genome of the novel strain MAH-18^T^. The G+C content of the genomic DNA of strain was 72.2 mol% and the predominant isoprenoid quinone was MK-8 (H_4_). The main polar lipids were phosphatidylglycerol, diphosphatidylglycerol, phosphatidylethanolamine, and unknown phospholipids. The major cellular fatty acids were determined to be C_16:0_ iso and C_17 : 1_* ω*6*c*. The DNA–DNA hybridization results and phenotypic, genotypic, and chemotaxonomic data demonstrated that strain MAH-18^T^ represents a novel species, for which the name *Nocardioides agri* sp. nov. is proposed, with MAH-18^T^ as the type strain (=KACC 19744^T^=CGMCC 1.13656^T^).

## Introduction

The genus *Nocardioides* of the family *Nocardioidaceae*, belongs to the phylum *Actinomycetota* and was first described by Prauser [1]. Members of the genus *Nocardioides* have been isolated from various habitats, such as soil, water, plant roots, oil shale columns, meadows, polluted environments, marine sediments, crustacean, alpine glacier cryoconite, halophytes, sea urchins, Roman catacombs, Antarctica, desert, forest, dandelion, sewage sludge, and caves [2,8]. Many of the novel *Nocardioides* species have been isolated from various regions of Korea [6]. At the time of writing, the genus *Nocardioides* includes 166 species with validly published names (https://lpsn.dsmz.de/genus/nocardioides). The common characteristics of the members of genus *Nocardioides* include Gram-stain-positive, aerobic, rod or cocci-shaped, and motile or non-motile cells with high DNA G+C content, in a range of 68.7–74.9 mol% [2,8]. Members of this genus contain menaquinone MK-8 (H_4_) as the predominant isoprenoid quinone. The major fatty acid of the members of this genus was determined to be C_16:0_ iso [2,8]. Bacteria have numerous beneficial uses for human welfare [9,10]. Members of the genus *Nocardioides* have various applications including the production of valuable enzymes, antibiotics, biodegradation of hazardous chemicals, and plant growth-promoting activities, etc [2,4,8, 11]. In the present study, we describe a novel bacterial species of genus *Nocardioides*, *Nocardioides agri* sp. nov., represented by MAH-18^T^, isolated from soil sampled in a garden. The purpose of this study is to clarify the taxonomic position of strain MAH-18^T^ in detail based on phenotypic, chemotaxonomic, and genotypic analyses. Moreover, the genome sequence of novel strain MAH-18^T^ was sequenced and analysed for the presence of putative natural product BGCs (biosynthetic gene clusters). The availability of whole genome sequences and synthetic biology-inspired tools makes it possible to utilize these BGCs to develop new chemicals [12]. Our data revealed some BGCs in the genome of the isolated strain.

## Isolation and ecology

Strain MAH-18^T^ was isolated from soil sampled in a flower garden located in Anseong, Republic of Korea (37° 00′ 33″ N 127° 23′ 01″ E). Sterile 0.85 % (w/v) NaCl solution was used to make suspension of the soil sample and for making serial dilutions. Afterward, 100 µl of individual dilution was spread onto Reasoner's 2A (R2A) agar plates (Difco), and the agar plates were placed at 30 °C incubator for 3 days. Single colonies were purified by repeated streaking on fresh R2A agar plates and preserved as a suspension in R2A broth containing glycerol (25 %, v/v) at −80 °C. Based on 16S rRNA gene sequencing results, one isolate, MAH-18^T^, was selected for further study. Isolate MAH-18^T^ has been deposited to the Korean Agriculture Culture Collection (KACC) and the China General Microbiological Culture Collection Center (CGMCC). For the comparative study, the reference strains *Nocardioides pyridinolyticus* JCM 10369^T^, *Nocardioides hankookensis* KCTC 19246^T^, *Nocardioides aquiterrae* KACC 17266^T^, *Nocardioides soli* KACC 17152^T^, *Nocardioides conyzicola* KCTC 29121^T^, and *Nocardioides mangrovi* JCM 34553^T^ were included and tested using the same laboratory conditions.

## 16S rRNA gene sequence and phylogenetic analysis

Extraction of the genomic DNA was achieved using a commercial genomic DNA extraction kit (Solgent). The extracted genomic DNA sample was used for the amplification of the 16S rRNA gene of strain MAH-18^T^. For PCR amplification, the bacterial universal primer pair (27 F-1492R) [13] was used. The PCR products were purified and sent to Solgent Co. Ltd. (Daejeon, Republic of Korea) for sequencing. Afterward, the 16S rRNA gene sequencing result of strain MAH-18^T^ was blasted in EzTaxon-e server (https://eztaxon-e.ezbiocloud.net/ [14]) and the 16S rRNA gene sequences of closely related species were analysed. The multiple alignments were accomplished using the clustal_x program [15] and the gaps were edited using the BioEdit program [16]. The evolutionary distances were calculated using the Kimura two-parameter model [17]. Phylogenetic trees were created using both the neighbour-joining method [18] and the maximum-likelihood method in the mega11 program [19], with bootstrap values based on 1000 replications [20].

**Fig. 1.**
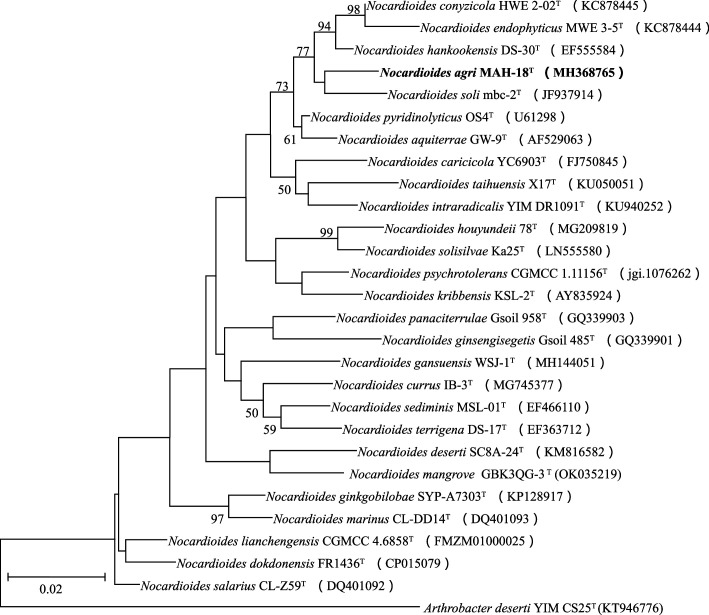
The maximum-likelihood tree based on 16S rRNA gene sequence analysis showing phylogenetic relationships of strain MAH-18^T^ and members of the genus *Nocardioides*. Bootstrap values less than 50 % based on 1000 replications are not shown at branching points. *Arthrobacter deserti* YIM CS25^T^ was used as an outgroup. Scale bar, 0.02 substitutions per nucleotide position.

The 16S rRNA gene sequence of strain MAH-18^T^ was 1404 bp. Based on the EzTaxon-e-server analysis using 16S rRNA gene sequences, the close relatives of strain MAH-18^T^ were *N. pyridinolyticus* OS4^T^ (97.9 %), *N. hankookensis* DS-30^T^ (97.9 %), * N. aquiterrae* GW-9^T^ (97.6 %), *N. soli* mbc-2^T^ (97.5 %), *N. conyzicola* HWE 2-02^T^ (97.4 %), and *N. mangrovi* GBK3QG-3^T^ (96.3 %). The relationship between strain MAH-18^T^ and other members of the genus *Nocardioides* was supported by the phylogenetic trees (Figs 1 and S1, available in the online version of this article). The maximum-likelihood tree showed that strain MAH-18^T^ is clustered within the genus *Nocardioides* (Fig. 1). This maximum-likelihood tree was also supported by the tree reconstructed by the neighbour-joining algorithm (Fig. S1) with a high bootstrap value. The phylogenetic analysis revealed that strain MAH-18^T^ is clearly grouped within the genus *Nocardioides*.

## Genome features and comparative analyses

The genomic DNA was extracted using a genomic DNA extraction kit (Solgent). The draft genome sequence of strain MAH-18^T^ was determined using an Illumina HiSeq X Ten and was assembled by SOAPdenovo version 3.10.1. The genome annotation was done using the NCBI Prokaryotic Genome Annotation Pipeline. The phylogenetic tree was also reconstructed using whole-genome sequences based on multi-locus sequence analysis (https://automlst.ziemertlab.com/analyze) [21]. To determine the degree of pairwise relatedness between MAH-18^T^ and the closest type strains, blast-based average nucleotide identity (ANI) was calculated as described by Yoon *et al*. [22]. While the digital DNA–DNA hybridization (dDDH) values were determined using the Genome-to-Genome Distance Calculator (http://ggdc.dsmz.de/ggdc.php) according to Meier-Kolthoff *et al*. [23]. For a whole genome-based taxonomic analysis, the genome sequence data were uploaded to the Type (Strain) Genome Server (TYGS; https://tygs.dsmz.de). The Genome blast Distance Phylogeny approach (GBDP) was also used to calculate dDDH values and reconstruct phylogenetic trees using TYGS [24,25]. CGView (http://cgview.ca/) was used for the schematic representation of the circular chromosome using the genome of strain AH-18^T^. The distribution of genes in the genome of strain MAH-18^T^ was investigated using the rast server [26].

**Fig. 2.**
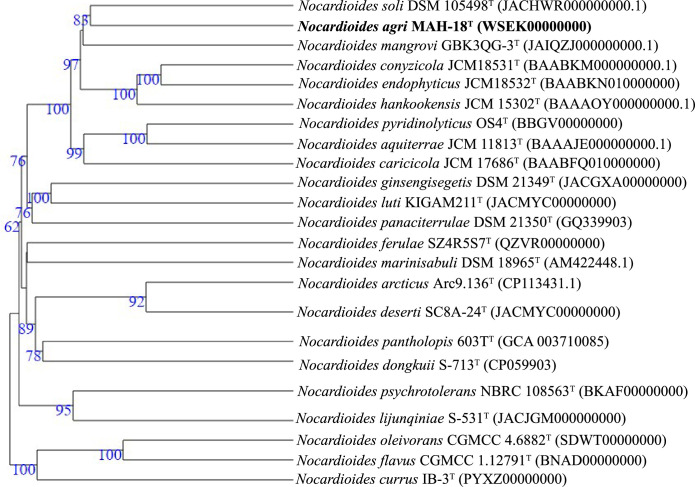
Whole-genome sequence-based GBDP (Genome blast Distance Phylogeny) tree showing the relationships of strain MAH-18^T^ with other closest type species. Tree inferred with FastME 2.1.6.1 from GBDP distances calculated from genome sequences. The branch lengths are scaled in terms of GBDP distance formula d5. The numbers above branches are GBDP pseudo-bootstrap support values >60 % from 100 replications, with an average branch support of 96.5 %. The tree was rooted at the midpoint.

**Fig. 3.**
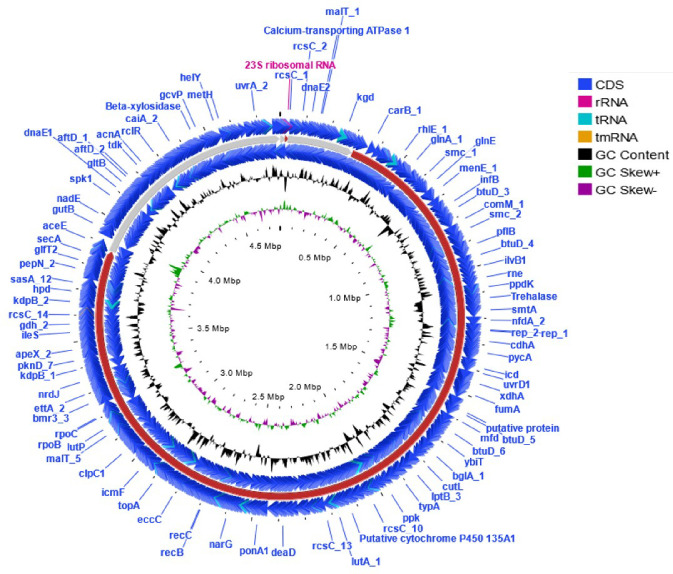
Schematic representation of the circular chromosome of novel strain MAH-18^T^, created by CG View server (http://cgview.ca/). Circle 1 (outermost) displays the contigs while circle 2 displays the G+C content plot and circle 3 (innermost) displays the GC skew. To indicate genome size inside and outside, the ruler was used in the chromosome map.

The draft genome sequence of strain MAH-18^T^ yielded a genome of 4.78 Mb after assembly, producing eight contigs with an N50 value of 3 568 698. Gene prediction allowed the annotation of 4572 protein-coding genes with 46 tRNA and three rRNA genes. The genomic DNA G+C content of strain MAH-18^T^ was directly calculated from its genome sequence and determined as 72.2 mol%, which lies in the expected range of genus *Nocardioides* [3,8]. Genome sequence features of strain MAH-18^T^ are shown in Table S1. The ANI (average nucleotide identity) values between strain MAH-18^T^ and *N. pyridinolyticus* OS4^T^, *N. hankookensis* DS-30^T^, *N. aquiterrae* GW-9^T^, *N. soli* mbc-2^T^, *N. conyzicola* HWE 2-02^T^, and *N. mangrovi* GBK3QG-3^T^ were 81.5, 82.9, 82.8, 83.4, 82.5, and 83.0 %, respectively (Table S2). The dDDH values based on the draft genomes between strain MAH-18^T^ and *N. pyridinolyticus* OS4^T^, * N. hankookensis* DS-30^T^, *N. aquiterrae* GW-9^T^, *N. soli* mbc-2^T^, *N. conyzicola* HWE 2-02^T^, and * N. mangrovi* GBK3QG-3^T^ were 24.4, 25.1, 25.0, 25.8, 24.6, and 25.0 %, respectively (Table S2). These ANI values and dDDH values are well below than the species threshold of 95–96 and 70 %, respectively, suggesting that MAH-18^T^ represents a novel species [27,29]. The phylogenetic tree that was reconstructed from multi-locus sequence analysis of whole-genome sequences showed that strain MAH-18^T^ is clustered with the members of genus *Nocardioides* (Fig. S2). Using the Genome blast Distance Phylogeny (GBDP) method and tree builder service, the phylogeny tree of strain MAH-18^T^ using its whole genome sequence were created. The GBDP phylogenetic tree reconstructed by using the whole genome indicated that strain MAH-18^T^ is clustered with the members of the genus *Nocardioides* (Fig. 2). Based on dDDH and ANI values and phylogenetic analysis, it is evident that strain MAH-18^T^ represents a novel species belonging to the genus *Nocardioides*. Fig. 3 shows the circular chromosomes based on the genome sequence of strain MAH-18^T^ using the CGView server (http://cgview.ca/) [30]. The rast functional annotations of the draft genome of strain MAH-18^T^ showed that 132 of the genes were involved with protein metabolism, 290 genes were associated with the metabolism of amino acids and derivatives, 90 genes were involved with DNA metabolism, 273 genes were linked with carbohydrate metabolism, and 167 genes were involved with the metabolism of vitamins, cofactors, and pigments. Moreover, the genome of strain MAH-18^T^ revealed 34 gene clusters for stress response and 84 genes for respiration (Table S3). The genome of strain MAH-18^T^ has five genes for motility and chemotaxis (Table S3). The presence of genes for flagellar motility and the presence of flagella (Fig. S3) showed that the phenotypic and genomic results are consistent with each other.

## Secondary metabolite biosynthetic gene cluster prediction

As a main approach for finding and annotating genes in BGCs across the genome, antiSMASH 7 [31] combined with ClusterBlast, ActiveSiteFinder, ClusterBlast, Cluster PFam analysis, and SubClusterBlast [31] were used for the discovery of BGCs in the genome of strain MAH-18^T^ for secondary metabolites. bagel4 was used for the prediction of bacteriocin [32].

Using antiSMASH 7.0, we found several BGCs on the genome of strain MAH-18^T^ for different secondary metabolites. Through the prediction using antiSMASH 7.0, five BGCs were discovered in the genome of strain MAH-18^T^ (Table 1). The BGCs for terpene, lassopeptide, redox-cofactor, etc. were discovered in the genome of strain MAH-18^T^ (Table 1). Among these BGCs, only one BGC of strain MAH-18^T^ (cluster 2, region 4.2) was 33 % identical to known BGCs. Other BGCs exhibited no similarity to previously identified BGCs. Moreover, bacteriocins and other ribosomally synthesized and post-translationally modified peptides (RiPPs) were discovered in the genome of strain MAH-18^T^ using bagel4 (Table 2). Condensed genes present in the genome of strain strain MAH-18^T^ with their functions are shown in Supplementary Materials 2.

**Table 1.** The analysis of biosynthetic pathways in *Nocardioides agri* MAH-18^T^ by antiSMASH 7.0

**Cluster serialno.**	**Region**	**Type**	**From**	**To**	**Most similar known cluster**	**Similarity**
1	Region 4.1	Other	629 110	669 763		
2	Region 4.2	Terpene	953 338	974 219	Carotenoid, terpene	33 %
3	Region 4.3	RRE-containing, lassopeptide	1 322 799	1 344 816		
4	Region 4.4	RRE-containing	1 630 310	1 649 940		
5	Region 4.5	Redox-cofactor	2 339 192	2 361 844		

**Table 2.** Clusters in the genome of strain MAH-18^T^ for RiPPs predicted by bagel4

No. of areas of interest (AOIs)	Start	End	**Class**
MAH-18_5.2.AOI_01	744647	764647	Sactipeptides
MAH-18_4.4.AOI_01	2339516	2359516	Sactipeptides

## Physiology and chemotaxonomy

The colony morphology was investigated after 3 days of incubation at 30 °C on R2A agar plate. Cell morphology was observed at ×11 000 magnification with a transmission electron microscope (JEM1010, jeol) using cells grown for 3 days at 30 °C on R2A agar. Briefly, the suspended cells were placed on carbon- and formvar-coated nickel grids for 30 s, and grids were floated on one drop of 0.1 % (w/v) aqueous uranyl acetate, blotted dry and then viewed with the transmission electron microscope under standard operating conditions. The motility assay was performed with the use of sulphide-indole-motility medium (Difco). The bioMérieux Gram-stain kit was used for the investigation of the Gram-stain reaction. This assay was performed according to the manufacturer’s instructions. The best growth medium was selected using several bacterial media such as R2A (Difco), nutrient agar, MacConkey agar (Oxoid), tryptone soya agar (Oxoid), and Luria–Bertani agar (Oxoid). Growth at various temperatures such as 4, 10, 15, 20, 25, 28, 30, 35, and 40 °C and different pH conditions from 4.0 to 10.0 (with 0.5 pH unit intervals) were investigated in R2A broth. During the pH test, acetate buffer was used for 4.0 to 6.5, while phosphate buffer was utilized for pH 7.0–10.0. R2A broth medium was employed for the salinity test, where the range of NaCl was 0–9 % NaCl (w/v) with 0.5 % intervals. The growth of the cell was determined by observing the optical density (OD) at 600 nm. To inspect anaerobic growth, the isolate was grown on R2A agar plates and incubated in a closed chamber with AnaeroGen kit (Thermo Scientific) for 7 days at 30 °C [33]. Synthesis of flexirubin-type pigments was tested using an aqueous 20 % KOH solution and monitored by changing the colour of the colony to red, purple or brown [34]. Catalase and oxidase activities were examined using 3 % (v/v) H_2_O_2_ and 1 % (w/v) *N*, *N*, *N*′,*N*′-tetramethyl-1, 4-phenylenediamine reagent (Sigma) respectively, as described by Huq [35]. Nitrate reduction was checked in nitrate broth containing 0.2 % KNO_3_ [35]. Indole production was examined using Kovacs reagent in 1 % tryptone broth [36]. Urease activity was analysed in Christensen’s medium [37]. The hydrolysis activity of following substrates was checked using R2A agar as the basal medium: 0.5 % l-tyrosine (Sigma), 0.1 % aesculin (0.02 % ferric citrate, Difco), casein (2.0 % skimmed milk, Oxoid), Tween 80 (0.01 % CaCl_2_.2H_2_O and 1.0 % Tween 80, Sigma), 12.0 % gelatin (Sigma), Tween 20(0.01 % CaCl_2_.2H_2_O and 1.0 % Tween 20, Sigma), 1.0 % starch (Difco), and DNA (DNase agar, Scharlau). Strain MAH-18^T^ was cultured on agar plates for 7 days at 30 °C. Afterward, the DNase activity was inspected by flooding the plates with 1 N HCl. Enzyme activities as well as carbon-source utilization of strain MAH-18^T^ along with the reference strains were performed using API ZYM and API 20NE test kits, respectively, according to the instructions of the manufacturer (bioMérieux).

Strain MAH-18^T^ is an aerobic, motile, Gram-positive, and rod-shaped organism with 0.4–0.7 µm wide and 0.9–2.0 µm long cells (Fig. S3). The colonies were beige in colour, smooth, spherical, and 0.1–0.4 mm in diameter when grown for 3 days. The following enzyme activities were positive: alkaline phosphatase, acid phosphatase, trypsin, leucine arylamidase, esterase lipase (C8), esterase (C4), β-glucosidase, α-glucosidase, and β-galactosidase. The morphological, physiological, and biochemical characteristics of strain MAH-18^T^ and related type strains were summarized in Table 3 and the species description.

**Table 3.** The biochemical and physiological characteristics of strain MAH-18^T^ and the reference strains of the genus *Nocardioides* Strains: 1, MAH-18^T^; 2, *N. hankookensis* KCTC 19246^T^; 3, *N. pyridinolyticus* JCM 10369^T^; 4, *N. aquiterrae* KACC 17266^T^; 5, *N. soli* KACC 17152^T^; 6, *N. conyzicola* KCTC 29121^T^ and 7, *N. mangrovi* JCM 34553^T^. All data were obtained from this work. All strains are aerobic, positive for catalase, hydrolysis of aesculin and gelatin. All strains are negative for *N*-acetyl-*β*-glucosaminidase, *α*-mannosidase, *α*-galactosidase, *α*-fucosidase, assimilation of capric acid and phenylacetic acid. +, Positive; w+, weakly positive; −, negative.

Characteristics	1	2	3	4	5	6	7
Isolation source	Garden soil	Soil	Oil-shale column	Groundwater	Soil	Plant root	Plant root
Cell morphology	Rod	Rod	Rod, cocci	Rod, cocci	Rod	Rod	Rod
Colony colour	Beige	White	Cream	Cream	Cream	Whitish	Greenish yellow
Oxidase	−	+	−	+	−	+	−
Reduction of nitrate (API 20 NE)	+	−	+	+	+	+	−
Hydrolysis of:							
Casein	−	+	+	+	+	+	+
Starch	−	+	+	−	+	+	w+
Urea (API 20 NE)	−	−	−	−	+	w+	−
Enzyme activity (API ZYM):							
Esterase (C4)	+	+	−	−	−	w+	+
Alkaline phosphatase	+	+	+	−	−	+	+
Esterase lipase (C8)	+	+	+	+	−	+	+
Lipase (C14)	w+	−	−	−	−	w+	−
Acid phosphatase	+	+	+	+	−	+	+
Leucine arylamidase	+	+	+	+	−	+	+
Valine arylamidase	w+	−	w	−	−	−	+
Cystine arylamidase	w+	−	−	w	+	w+	+
Trypsin	+	−	+	+	−	+	+
*α*-Chymotrypsin	w+	−	−	−	−	−	−
*β*-Glucuronidase	w+	−	−	−	−	−	−
Naphthol-AS-BI-phosphohydrolase	w+	+	+	+	−	+	+
*α*-Glucosidase	+	−	+	+	−	+	+
*β*-Glucosidase	+	−	−	w+	−	+	+
*β*-Galactosidase	+	−	−	−	−	w+	+
Assimilation of (API 20 NE):							
d-Glucose	−	+	+	+	+	−	+
l-Arabinose	−	+	−	−	+	−	−
*N*-Acetyl-glucosamine	−	−	−	−	−	+	w+
Maltose	−	+	+	+	+	−	+
Gluconate	−	w	−	+	+	+	w+
Adipic acid	−	+	+	−	−	w+	w+
Malic acid	−	+	−	−	+	−	w+
DNA G+C content (mol%)	72.2	63.8	71.7	72.2	71.7	71.5	72.2

For fatty acid analysis, strain MAH-18^T^ and the reference strains were cultured on agar plates for 3 days at 30 °C, and afterward, the cells were collected. Sherlock Microbial Identification System (midi) method was followed for the extraction, methylation as well as saponification of the fatty acids, and extracted samples were examined by capillary GC [38]. The respiratory quinones of strain MAH-18^T^ were extracted from freeze-dried cells according to Collins [39]. Then, the purified quinone was analysed by the method of Collins and Jones [40] using HPLC (Alliance 2690 system, Waters). Polar lipids were extracted from dried cells (100 mg) according to the method described by Minnikin *et al*. [41] with modifications. Briefly, 100 mg freeze-dried cells were suspended in 2 ml of 0.3 % saline in a screw-capped tube. Then, 10 ml methanol was added and heated at 100 °C for 5 min. After cooling, 5 ml chloroform and 3 ml saline were added and kept in a shaker for 3 h. Then, the supernatant was collected through centrifugation at 5000 r.p.m. for 10 min. 5 ml each of chloroform and saline were added to the supernatant. Finally, the down layer (chloroform layer) was collected and concentrated to dryness. The extracted polar lipids were analysed by two-dimensional thin-layer chromatography (TLC) using TLC Kiesel gel 60F254 (Merck) plates (10×10 cm). The total polar lipids, aminolipids, glycolipids, and phospholipids were detected by staining the plates with 5 % molybdophosphoric acid, 0.2 % ninhydrin, 15 % α-naphthol (dissolved in 95 % ethanol) and molybdenum blue, respectively [42].

Cellular fatty acids of strain MAH-18^T^ and the related *Nocardioides* type strains are shown in Table 4. The major cellular fatty acids of strain MAH-18^T^ were determined to be C_16:0_ iso (41.5 %) and C_17 : 1_* ω*6*c* (15.3 %). The novel strain MAH-18^T^ also contained considerable amounts of C_18 : 1_* ω*9*c* (7.5 %), C_14:0_ iso (5.7 %), and C_16:0_ iso (5.0 %). The reference type stains of the genus *Nocardioides* revealed similar fatty acid profiles, but there were remarkable quantitative differences when cultivated under the same conditions (Table 4). The major respiratory quinone detected in strain MAH-18^T^ was menaquinone, MK-8 (H_4_), one of the common characteristics in the genus *Nocardioides*. The cellular polar lipids of strain MAH-18^T^ consisted of phosphatidylethanolamine, diphosphatidylglycerol, phosphatidylglycerol, one unknown phospholipid, and one unknown lipid (Fig. S4). The similar major polar lipids were detected in close relatives of the genus *Nocardioides* [3].

**Table 4.** Fatty acid profiles of strain MAH-18^T^ and the reference strains of genus *Nocardioides* Strains: 1, MAH-18^T^; 2, *N. hankookensis* KCTC 19246^T^; 3, *N. pyridinolyticus* JCM 10369^T^; 4, *N. aquiterrae* KACC 17266^T^; 5, *N. soli* KACC 17152^T^; 6, *N. conyzicola* KCTC 29121^T^ and 7, *N. mangrovi* JCM 34553^T^. All data were obtained from this work. Fatty acid percentages amounting to less than 1 % of the total fatty acids in all strains were not included in table. tr, Trace (less than 1.0 %); nd, not detected.

Fatty acid	1	2	3	4	5	6	7
C_14:0_ iso	5.7	2.2	1.0	1.5	1.3	3.3	1.4
C_15:0_ iso	5.0	6.8	6.3	2.6	7.2	nd	7.2
C_15 : 0_ anteiso	3.4	tr	tr	tr	1.2	nd	1.5
C_16 : 0_	1.8	2.6	3.8	tr	1.5	3.7	2.2
C_16 : 0_ iso	41.5	44.7	53.0	68.8	59.5	60.2	35.5
C_16 : 1_ iso H	tr	tr	2.8	2.7	1.4	nd	nd
C_17 : 0_	4.2	1.9	tr	tr	tr	1.8	1.3
C_17:0_ iso	1.1	4.7	2.5	tr	1.6	nd	9.7
C_17:0_ anteiso	2.3	2.8	7.9	4.7	4.9	2.4	14.4
C_17 : 0_ 10-methyl	1.6	3.1	1.9	3.6	5.8	2.6	1.4
C_17 : 1_* ω*8*c*	15.3	6.2	tr	tr	1.4	nd	1.9
C_18 : 0_	tr	1.0	2.7	1.3	tr	4.2	3.0
C_18:0_ iso	tr	1.2	tr	1.4	tr	3.0	2.8
C_18 : 1_* ω*9*c*	7.5	10.7	1.7	1.5	2.9	6.1	7.8
C_18 : 0_ 10-methyl	1.7	3.8	2.6	5.4	2.7	6.6	5.6
Summed feature 6*	4.2	1.2	nd	nd	nd	nd	nd
Summed feature 9†	tr	1.3	5.4	4.2	3.1	1.1	1.2

*Summed feature 6 contained C_19 : 1_* ω*11*c* and/or C_19 : 1_* ω*9*c*.

†Summed feature 9 contained C_17:1 iso_ *ω*9*c* and/or C_16 : 0_ 10-methyl.

In summary, the characteristics of strain MAH-18^T^ were consistent with descriptions of the genus *Nocardioides* in terms of physiological, morphological, biochemical, and chemotaxonomic properties. The results of this study indicated that strain MAH-18^T^ should be assigned to the genus *Nocardioides* as a novel species, for which the name *Nocardioides agri* sp. nov. is proposed.

## Description of *Nocardioides agri* sp. nov.

*Nocardioides agri* (a’gri. L. gen. n. *agri*, of a field).

Cells are Gram-stain-positive, motile, aerobic, and rod-shaped (0.4–0.7 µm wide and 0.9–2.0 µm long). Colonies on R2A agar are beige in colour, smooth, spherical, and 0.1–0.4 mm in diameter when grown for 3 days. The strain gives positive result for catalase and negative results for oxidase and flexirubin-type pigments. Cell growth occurs on R2A agar and nutrient agar but not on Luria-Bertani agar, tryptone soya agar or MacConkey agar. Bacteria can grow on R2A agar at 20–40 °C with optimum growth at 30 °C. Strain MAH-18^T^ can grow in R2A broth at pH 6.0–8.0 (optimum, pH 7.0) and at 0–1.0 % NaCl (optimum, 0 % NaCl). Cells can hydrolyze aesculin, gelatin, and Tween 20 but not l-arginine, casein, l-tyrosine, starch, DNA, urea, and Tween 80. Nitrate reduction was positive but negative results were obtained for indole production and glucose fermentation. The following compounds are not utilized as sole carbon source: adipic acid, capric acid, d-glucose, maltose, d-mannitol, d-mannose, gluconate, l-arabinose, malic acid, *N*-acetyl-glucosamine, phenylacetic acid, and trisodium citrate (API 20 NE). Positive for the following enzyme activities: acid phosphatase, trypsin, alkaline phosphatase, leucine arylamidase, β-glucosidase, esterase (C4), α-glucosidase, esterase lipase (C8), and β-galactosidase; weakly positive for lipase (C14), cysteine arylamidase, valine arylamidase, α-chymotrypsin, naphtol-AS-BI-phosphohydrolase, and β-glucuronidase; but negative for *α*-mannosidase, *N*-acetyl-*β*-glucosaminidase, *α*-galactosidase, and α-fucosidase. The predominant isoprenoid quinone is MK-8 (H_4_) and the major cellular fatty acids are C_16:0 iso_ and C_17 : 1_* ω*6*c*. The main polar lipids were phosphatidylglycerol, diphosphatidylglycerol, phosphatidylethanolamine, and unknown phospholipid. The genomic DNA G+C content of type strain is 72.2 mol%.

The type strain, MAH-18^T^ (=KACC 19744^T^=CGMCC 1.13656^T^), was isolated from soil sample of a garden located in Anseong, Republic of Korea. The NCBI GenBank accession numbers for the 16S rRNA gene and draft genome sequences of strain MAH-18^T^ are MH368765 and WSEK00000000, respectively.

## supplementary material

10.1099/ijsem.0.006407 Uncited Supplementary Material 1.

## Abbreviations

ANI: average nucleotide identity
BGCs: biosynthetic gene clusters
dDDH: digital DNA–DNA hybridization
GBDP: genome blast distance phylogeny
RiPPs: ribosomally synthesized and post-translationally modified peptides
