# Supplementary material for: Nocardioides agri sp. nov., isolated from garden soil
Source: Int J Syst Evol Microbiol. 2024 Jun 18;74(6):006407. doi: 10.1099/ijsem.0.006407 (PMC11261691; doi:10.1099/ijsem.0.006407)
Supplement: Uncited Supplementary Material 1. [file ijsem-74-06407-s001.pdf]

Supplementary Material

Nocardioides agri sp. nov., isolated from garden soil.

Md. Amdadul Huq[1†*], Kihong NAM[2†], Md. Shahedur Rahman[3], M. Mizanur Rahman[4], Md. Anowar Khasru Parvez[5], Kwon-Kyoo Kang[2*], Shahina Akter[6],

[1] *Department of Food and Nutrition, Chung-Ang University, Anseong-si, Gyeonggi-do, 17546, Republic of Korea.*

[2] *Department of Horticultural Life Science, Hankyong National University, Anseong-si, Gyeonggi-do, 17579, Republic of Korea.*

[3] *Department of Genetic Engineering and Biotechnology, Jashore University of Science and Technology, Jashore 7408, Bangladesh.*

[4] *Department of Biotechnology and Genetic Engineering, Faculty of Biological Science, Islamic University, Kushtia-7003, Bangladesh.*

[5] *Department of Microbiology, Jahangirnagar University, Savar, Dhaka-1342, Bangladesh.*

[6] *Department of Food Science and Biotechnology, Gachon University, Seongnam, 461-701, Republic of Korea.*

† These authors contributed equally to this work.

[*] Corresponding author: Md. Amdadul Huq, E-mail: amdadbge@gmail.com, amdadbge100@cau.ac.kr

Kwon-Kyoo Kang, E-mail: kykang@hknu.ac.kr

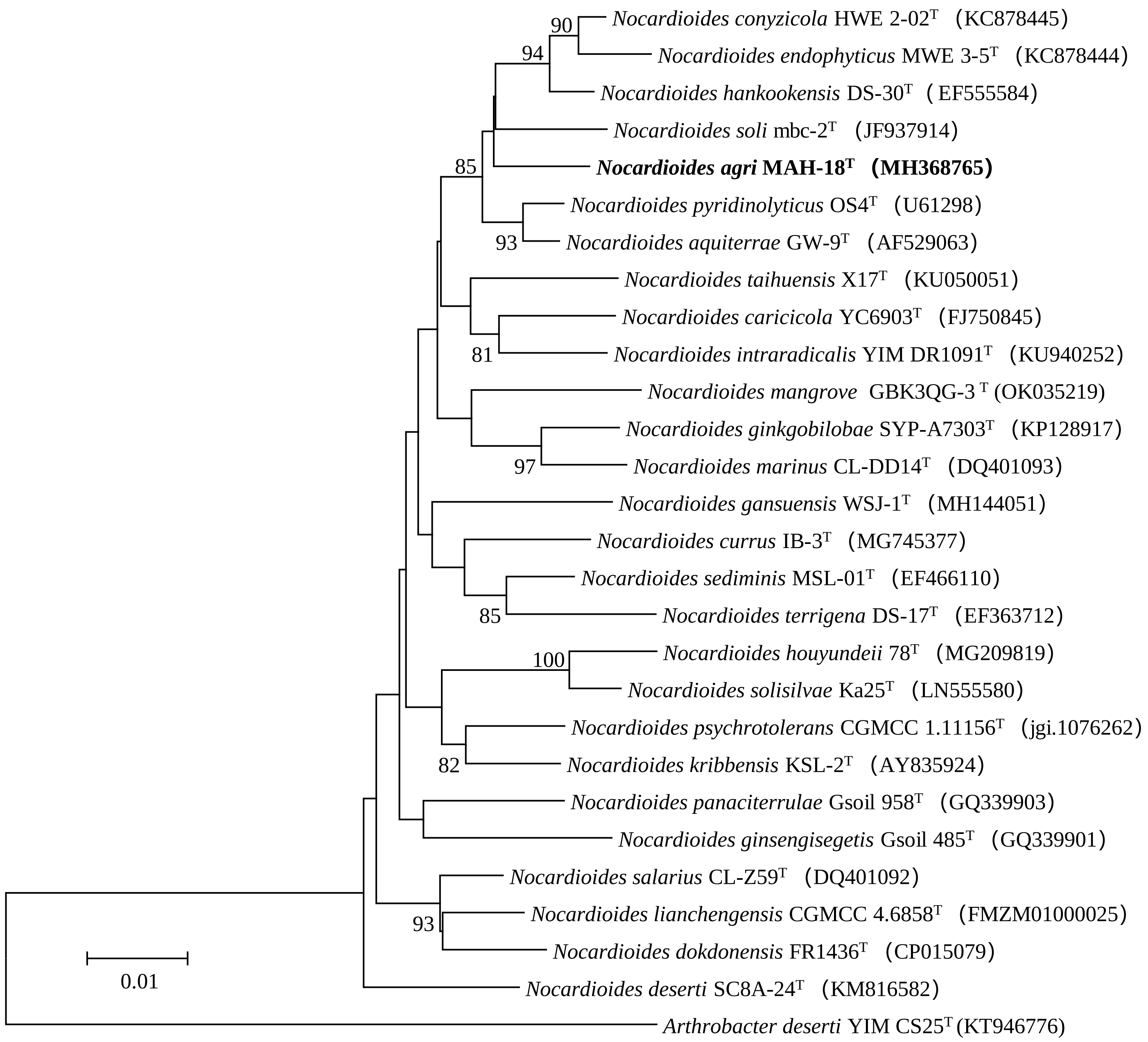

Supplementary Fig. S1. The neighbor-joining (NJ) tree based on 16S rRNA gene sequences, showing phylogenetic relationships of strain MAH-18^T and members of genus *Nocardioides*. Bootstrap values more than 70 % based on 1,000 replications are shown at branching points. Scale bar, 0.01 substitutions per nucleotide position.

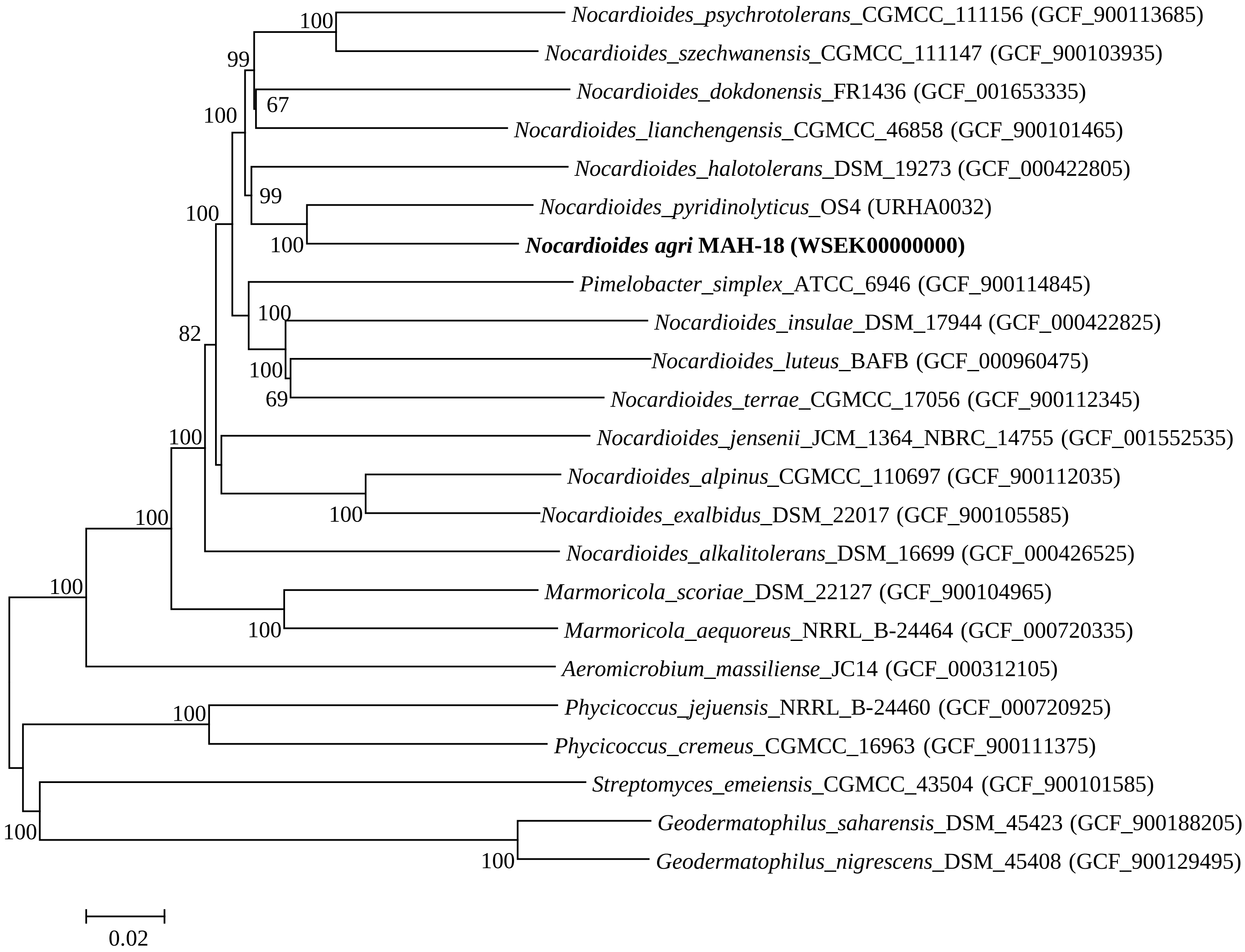

Supplementary Fig. S2. Phylogenetic tree constructed from a comparative analysis of whole genome sequences showing the relationships of strain MAH-18^T with other closest species. This tree was constructed via the Automated Multi-Locus Species Tree online web server, and with the Mega-7 program using the aligned sequences of Automated Multi-Locus Species analysis. A total of 89 genes were used to construct the tree. Bootstrap values (expressed as percentages of 1000 replications) greater than 50 % are shown at the branch points. The Bar represents 0.02 substitutions per nucleotide position.

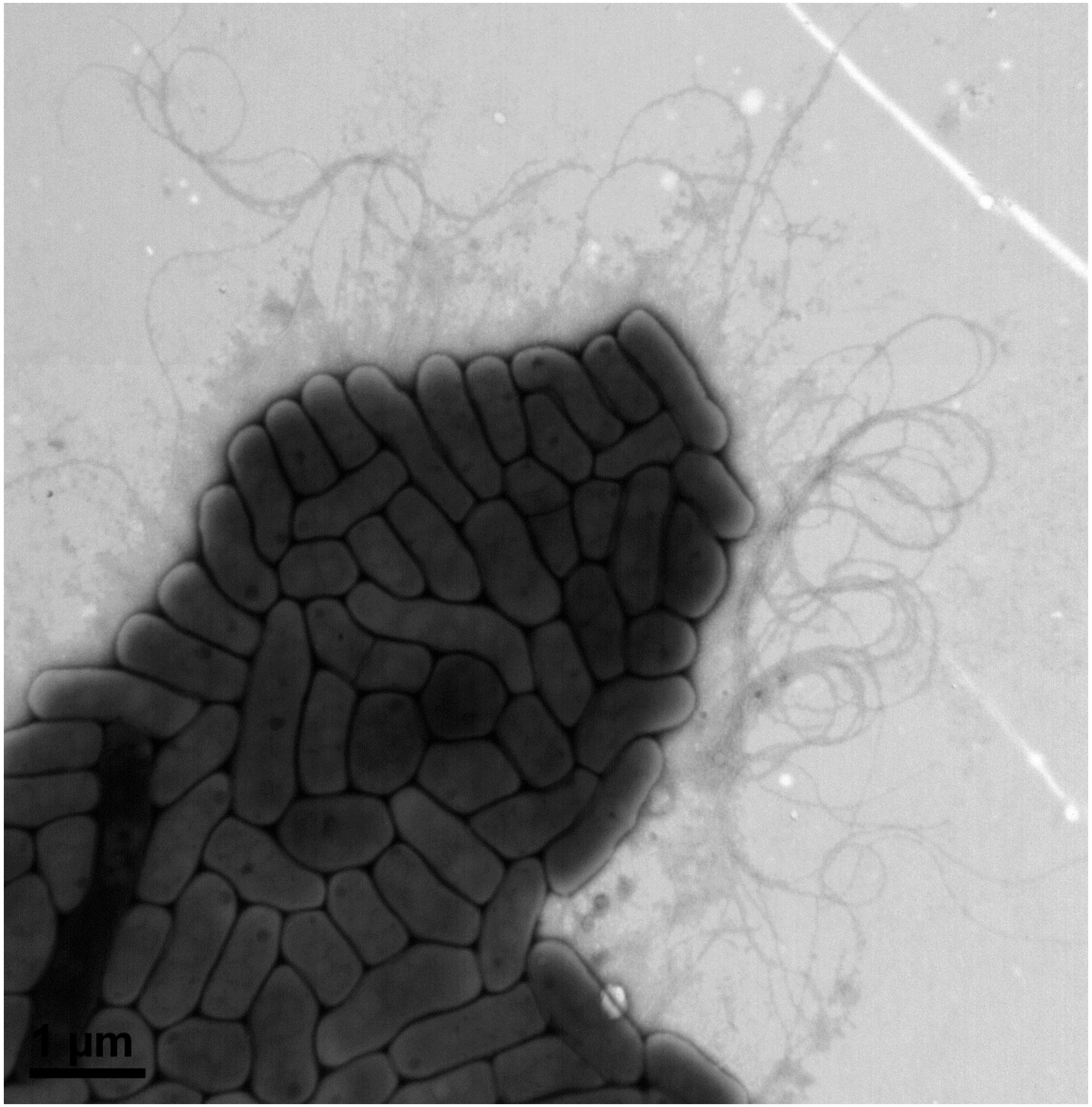

Supplementary Fig. S3 Transmission electron micrograph of strain MAH-18^T after negative staining with uranyl acetate, Bar, 1 µm.

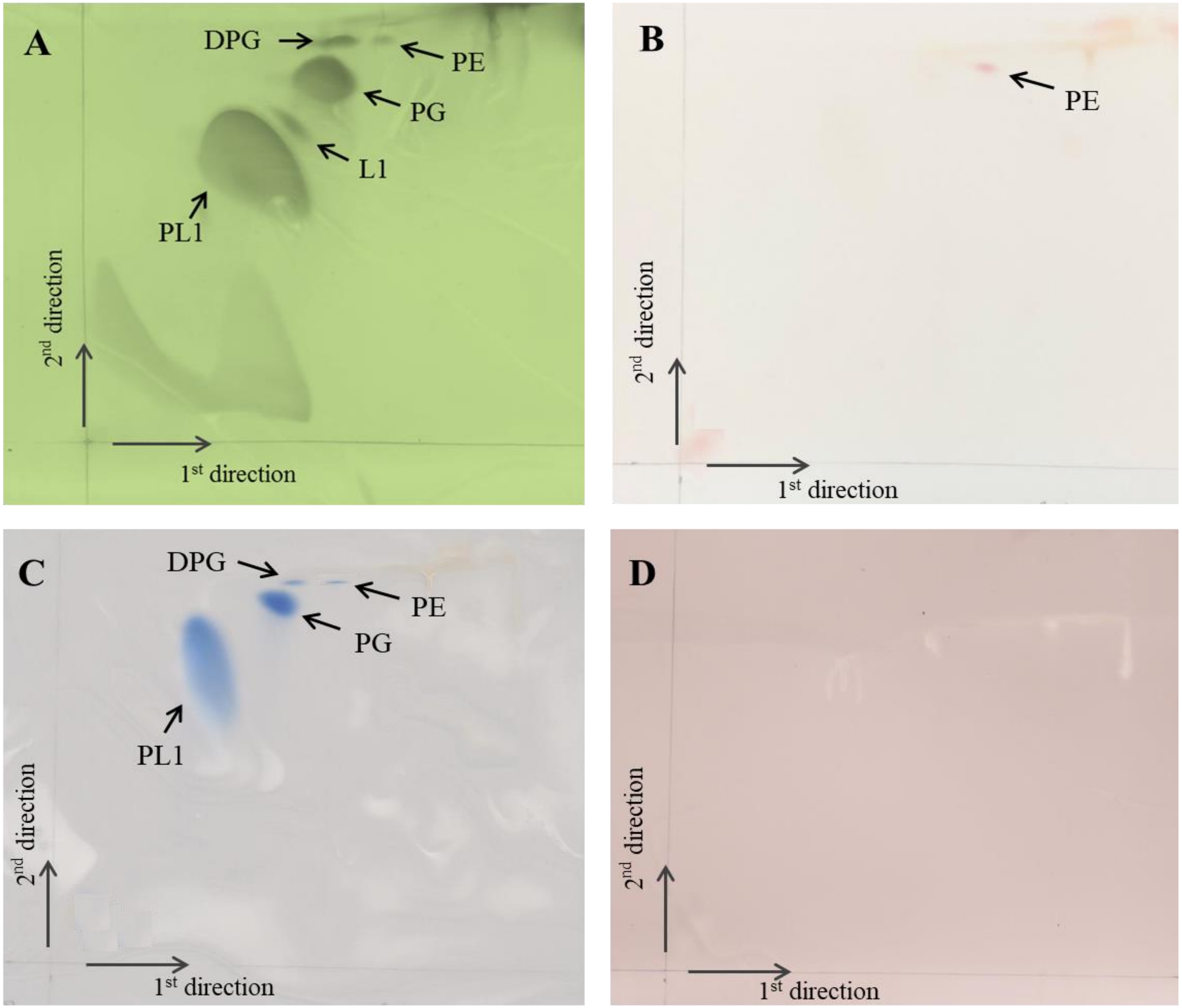

Supplementary Fig. S4. Two-dimensional TLC of the polar lipids of strain MAH-18^{T}. The samples were spotted on the corner of two-dimensional thin layer chromatography (2D-TLC) and developed in the first direction by using chloroform/methanol/water (65:25:4, by v/v/v) while in the second direction developed by chloroform/acetic acid/methanol/water (80:15:12:4, by v/v/v) as solvent systems. (A) Total lipids detected by spraying with 5% ethanolic molybdophosphoric acid; (B) Aminolipids detected by spraying with 0.2% (w/v) ninhydrin; (C) Phospholipids detected by spraying with molybdenum blue; and (D) Glycolipids were analysed by α naphthol reagent. Abbreviations: DPG, diphosphatidylglycerol; PG, phosphatidylglycerol; PE, phosphatidylethanolamine; PL1, unknown phospholipid; and L1, unknown lipid.

Supplementary Table S1. Genome sequence features of novel strain *Nocardioides agri* MAH-18^{T}.

**Features**	**Strain MAH-18^{T}**
NCBI Accession No.	WSEK00000000
Biosample	SAMN13483044
BioProject	PRJNA593637
Genome coverage	156.0x
Completeness	99.62%
Contamination level	0.69%
Total sequence length (nt)	4,788,325
Number of contigs	8
Number of scaffolds	5
Scaffold N50	3,568,698
Scaffold L50	1
Sequencing method	de novo
Annotation pipeline	NCBI Prokaryotic Genome
DNA G+C content (mol%)	72.2
Total genes	4,662
Genes (coding)	4,572
Number of RNAs	52
tRNAs	46
rRNAs	3

Supplementary Table S2. dDDH and ANI values between the proposed novel strain *Nocardioides agri* MAH-18^{T} and the closest type strains.

Query genome	Reference genome	dDDH value				ANI (%)
		DDH	Model C.I.	Distance	Prob. DDH >= 70%	
Nocardioides agri MAH-18^{T} (WSEK00000000)	*Nocardioides soli* DSM 105498^{T} (JACHWR000000000.1)	25.8%	[23.5 - 28.3%]	0.1682	0.01%	83.4
	Nocardioides hankookensis JCM 15302^{T} (BAAAOY000000000.1)	25.1%	[22.7 - 27.5%]	0.1737	0.01%	82.9
	Nocardioides mangrovi GBK3QG-3^{T} (JAIQZJ000000000.1)	25.0%	[22.6 - 27.4%]	0.1745	0.01%	83.0
	Nocardioides aquiterrae JCM 11813^{T} (BAAAJE000000000.1)	25.0%	[22.7 - 27.5%]	0.1742	0.01%	82.8
	Nocardioides conyzicola JCM 18531^{T} (BAABKM000000000.1)	24.6%	[22.2 - 27%]	0.1776	0.01%	82.5
	Nocardioides pyridinolyticus OS4^{T} (BBGV00000000)	24.4%	[21.1 – 26.9%]	0.1789	0.01%	81.5

Supplementary Table S3. Distribution of genes based on COG functional categories in the genome of novel strain *Nocardioides agri* MAH-18^{T}.

**COG Function**	***Nocardioides agri***
Cofactors, vitamins, prosthetic groups, pigments	167
Cell wall and capsule	31
Virulence, disease and defense	25
Potassium metabolism	4
Photosynthesis	0
Miscellaneous	31
Phages, prophages, transposable elements, plasmids	5
Membrane transport	43
Iron acquisition and metabolism	5
RNA metabolism	39
Nucleosides and nucleotides	98
Protein metabolism	132
Motility and chemotaxis	5
Regulation and cell signaling	11
Secondary metabolism	0
DNA metabolism	90
Fatty acids, lipids, and isoprenoids	137
Nitrogen metabolism	22
Dormancy and sporulation	1
Respiration	84
Stress response	34
Metabolism of aromatic compounds	40
Amino acids and derivatives	290
Sulfur metabolism	12
Phosphorus metabolism	25
Carbohydrates	273

Supplementary Materials 2 *Nocardioides agri* **MAH-18**^{T}

MAH-18_4.4.AOI_01

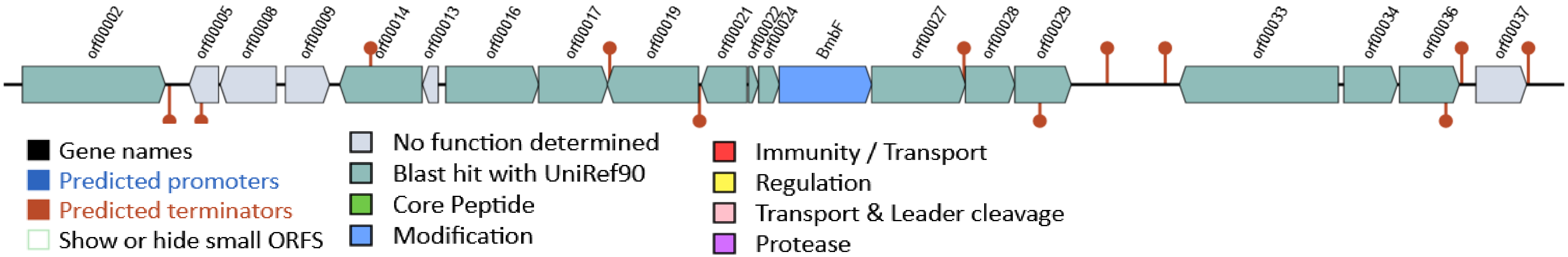

Name	Function	Motifs
orf00002	Phosphoenolpyruvate carboxykinase [GTP] OS=Hungateiclostridium thermocellum (strain ATCC 27405 / DSM 1237 / JCM 9322 / NBRC 103400 / NCIMB 10682 / NRRL B-4536 / VPI 7372) OX=203119 GN=pckG PE=3 SV=1	
orf00005		
orf00008		
orf00009		
orf00014	HTH-type transcriptional regulator IpsA OS=Corynebacterium glutamicum (strain ATCC 13032 / DSM 20300 / JCM 1318 / LMG 3730 / NCIMB 10025) OX=196627 GN=ipsA PE=1 SV=1	
orf00013		
orf00016	Sulfoquinovose isomerase OS=Salmonella typhimurium (strain LT2 / SGSC1412 / ATCC 700720) OX=99287 GN=yihS PE=1 SV=1	
orf00017	Fructokinase OS=Rhizobium leguminosarum bv. trifolii OX=386 GN=frk PE=3 SV=1	
orf00019	Acyl-CoA dehydrogenase OS=Bacillus subtilis (strain 168) OX=224308 GN=acdA PE=2 SV=1	
orf00021	Putative mycofactocin biosynthesis transcriptional regulator MftR OS=Mycobacterium tuberculosis (strain ATCC 25618 / H37Rv) OX=83332 GN=mftR PE=1 SV=1	
orf00022	Putative electron carrier mycofactocin OS=Mycobacterium tuberculosis (strain ATCC 25618 / H37Rv) OX=83332 GN=mftA PE=4 SV=1	
orf00024	Putative mycofactocin system protein MftB OS=Mycobacterium tuberculosis (strain ATCC 25618 / H37Rv) OX=83332 GN=mftB PE=4 SV=2	
BmbF	Putative mycofactocin radical SAM maturase MftC OS=Mycobacterium tuberculosis (strain CDC 1551 / Oshkosh) OX=83331 GN=mftC PE=3 SV=1	Sac_1;PF04055
orf00027	Putative mycofactocin system heme/flavin oxidoreductase MftD OS=Mycobacterium tuberculosis (strain CDC 1551 / Oshkosh) OX=83331 GN=mftD PE=3 SV=1	
orf00028	Putative mycofactocin system creatinine amidohydrolase family protein MftE OS=Mycobacterium tuberculosis (strain ATCC 25618 / H37Rv) OX=83332 GN=mftE PE=1 SV=1	
orf00029	(-)-trans-carveol dehydrogenase OS=Rhodococcus erythropolis OX=1833 GN=limC PE=1 SV=1	
orf00033	Uncharacterized MFS-type transporter Rv1877 OS=Mycobacterium tuberculosis (strain ATCC 25618 / H37Rv) OX=83332 GN=Rv1877 PE=3 SV=1	
orf00034	F420-dependent NADP reductase OS=Methanothermobacter thermautotrophicus (strain ATCC 29096 / DSM 1053 / JCM 10044 / NBRC 100330 / Delta H) OX=187420 GN=fno PE=1 SV=1	
orf00036	Carnitinyl-CoA dehydratase OS=Proteus mirabilis (strain HI4320) OX=529507 GN=caiD PE=3 SV=1	
orf00037		

Supplementary Materials 2 *Nocardioides agri* MAH-18^{T}

MAH-18_5.2.AOI_01

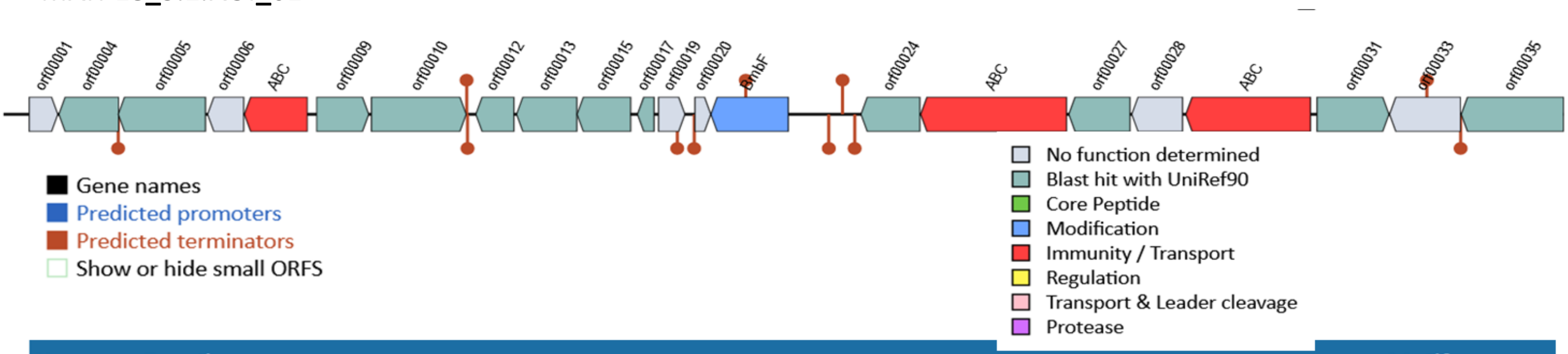

Name	Function	Motifs
orf00001		
orf00004	Probable membrane transporter protein YfcA OS=Escherichia coli (strain K12) OX=83333 GN=yfcA PE=1 SV=1	
orf00005	Uncharacterized protein Rv1488 OS=Mycobacterium tuberculosis (strain ATCC 25618 / H37Rv) OX=83332 GN=Rv1488 PE=1 SV=1	
orf00006		
ABC	Uncharacterized ABC transporter ATP-binding protein YlmA OS=Bacillus subtilis (strain 168) OX=224308 GN=ylmA PE=2 SV=2	PF00005
orf00009	Uncharacterized protein ML1804 OS=Mycobacterium leprae (strain TN) OX=272631 GN=ML1804 PE=4 SV=1	
orf00010	Phosphoserine phosphatase OS=Streptomyces coelicolor (strain ATCC BAA-471 / A3(2) / M145) OX=100226 GN=SCO1808 PE=1 SV=1	
orf00012	Uncharacterized protein ML1117 OS=Mycobacterium leprae (strain TN) OX=272631 GN=ML1117 PE=3 SV=1	
orf00013	Enoyl-[acyl-carrier-protein] reductase [NADH] OS=Mycobacterium tuberculosis (strain CDC 1551 / Oshkosh) OX=83331 GN=inhA PE=3 SV=1	
orf00015	3-oxoacyl-[acyl-carrier-protein] reductase FabG OS=Mycolicibacterium smegmatis (strain ATCC 700084 / mc(2)155) OX=246196 GN=fabG PE=1 SV=2	
orf00017	Dodecin OS=Halorhodospira halophila (strain DSM 244 / SL1) OX=349124 GN=Hhal_0546 PE=1 SV=1	
orf00019		
orf00020		
BmbF	GTP 3',8-cyclase OS=Streptomyces coelicolor (strain ATCC BAA-471 / A3(2) / M145) OX=100226 GN=moaA PE=3 SV=1	PF04055
orf00024	Glucose 1-dehydrogenase 2 OS=Bacillus megaterium OX=1404 GN=gdhII PE=3 SV=1	
ABC	Lipid A export ATP-binding/permease protein MsbA OS=Syntrophus aciditrophicus (strain SB) OX=56780 GN=msbA PE=3 SV=1	PF00005
orf00027	3-hydroxypropionyl-coenzyme A dehydratase OS=Metallosphaera sedula (strain ATCC 51363 / DSM 5348 / JCM 9185 / NBRC 15509 / TH2) OX=399549 GN=Msed_2001 PE=1 SV=1	
orf00028		
ABC	Putative ATP-binding protein YdiF OS=Bacillus subtilis (strain 168) OX=224308 GN=ydiF PE=3 SV=2	PF00005;PF00005
orf00031	Uncharacterized protein MT2651 OS=Mycobacterium tuberculosis (strain CDC 1551 / Oshkosh) OX=83331 GN=MT2651 PE=4 SV=1	
orf00033		
orf00035	Multidrug efflux system permease protein Rv1217c OS=Mycobacterium tuberculosis (strain ATCC 25618 / H37Rv) OX=83332 GN=Rv1217c PE=1 SV=1	
